# The Hospital Pageant Meeting

**Published:** 1921-06-04

**Authors:** W. Parkes

**Affiliations:** Secretary. St. Mary's Hospital, Paddington W. 2


### THE EDITOR'S LETTER-BOX.

**Correspondence on all subjects is invited, but we cannot in any way be responsible for the opinions expressed by our correspondents, who must give their name and address as a guarantee of good faith, but not necessarily for publication. Correspondents are reminded that brevity of style and conciseness of statement greatly facilitate early insertion.**

### The Hospital Pageant Meeting.

*To the Editor of The Hospital.*

Sir,—I shall be glad if you will be so good as to correct an inaccuracy which appeared in your report of the meeting of the Hospital Secretaries to consider the question of the Hospital Pageant. I am afraid the remarks of another speaker have been attributed to me, for I neither made the suggestion that the decision of the meeting should be sent to the Committees of the London Hospitals, nor am I responsible for the suggestion that the co-operation of the large stores should be secured in the matter. Obviously the remarks of another speaker have been in error attributed to me.

Yours faithfully,
W. Parkes, Secretary.

St. Mary's Hospital, Paddington W. 2,
May 27, 1921.

[We regret the inaccuracy. The reporter had difficulty in getting the names of speakers, as they were not, we are informed, handed up to the Chair.—Ed., The Hospital.]

### An Appreciation.

*To the Editor of The Hospital.*

